# A Prototype-Guided 3D Deep Learning Framework for Myocardial Perfusion Scintigraphy Segmentation

**DOI:** 10.3390/jcm15135314

**Published:** 2026-07-07

**Authors:** Madallah Alruwaili, Mahmood A. Mahmood

**Affiliations:** 1 Department of Computer Engineering and Networks, College of Computer and Information Sciences, Jouf University, Sakaka 72388, Saudi Arabia; 2 Department of Information Systems, College of Computer and Information Sciences, Jouf University, Sakaka 72388, Saudi Arabia; mamahmood@ju.edu.sa

**Keywords:** myocardial perfusion imaging, single-photon emission computed tomography, SPECT, myocardial perfusion scintigraphy, 3D deep learning, medical image segmentation, anatomy-aware learning, prototype memory refinement

## Abstract

**Background**: Myocardial perfusion scintigraphy (MPS) is widely used for noninvasive assessment of coronary artery disease, but publicly available datasets suitable for reproducible deep learning segmentation studies remain limited. This paper proposes CardioProto-SegNet, an image-only 3D anatomy-directed segmentation framework for myocardial region delineation using the public Myocardial Perfusion Scintigraphy Image Database v1.0.0 from PhysioNet, which contains 83 patient studies. **Methods**: The model is implemented as a 3D U-Net-like residual encoder–decoder network enhanced with squeeze-and-excitation channel recalibration and compact prototype-memory refinement at the bottleneck. Because the public dataset does not provide structured clinical variables, all reported results correspond to image-only myocardium segmentation. **Results**: Experimental evaluation demonstrated reliable segmentation performance on the available public dataset. CardioProto-SegNet achieved a Dice score of 0.7402 on the holdout test split. In five-fold cross-validation, the model obtained a mean Dice of 0.8239, mean IoU of 0.6870, mean accuracy of 0.9943, mean ROC-AUC of 0.9867, and mean PR-AUC of 0.8561. Since confirmed ischemia or infarction labels were not available, an exploratory image-derived subgroup analysis was additionally performed based on myocardial ROI uptake heterogeneity to examine model behavior in lower- and higher-heterogeneity cases. The ablation study showed that residual connections were important for stable segmentation performance, while the deeper variant achieved the highest tested performance, with a Dice score of 0.8290, IoU of 0.7096, and PR-AUC of 0.8831. **Conclusions**: Overall, the findings suggest that CardioProto-SegNet provides a reproducible public dataset benchmark for myocardium segmentation in MPS and may serve as a foundation for future downstream quantitative and CAD-oriented analysis when larger datasets with clinical labels become available.

## 1. Introduction

Coronary artery disease (CAD) remains a major cause of morbidity and mortality worldwide, and noninvasive imaging plays an important role in diagnosis, risk stratification, and treatment planning. Myocardial perfusion imaging (MPI), particularly single-photon emission computed tomography (SPECT), is widely used to assess myocardial blood flow, ischemia, and perfusion defects related to infarction. Despite its clinical value, SPECT-MPI interpretation still depends on visual assessment and several processing steps, which may introduce inter-reader variability and reduce workflow efficiency in high-volume clinical settings. Deep learning has therefore gained increasing attention as a tool for improving automation, standardization, and decision support in nuclear cardiology [1,2].

Recent studies have shown the potential of deep learning for CAD assessment using MPI. Convolutional neural networks and explainable artificial intelligence models have demonstrated promising performance compared with conventional quantitative indicators such as defect size and total perfusion deficit. For example, Liu et al. reported improved diagnostic accuracy using deep learning for stress-only myocardial perfusion SPECT, while Otaki et al. showed that an explainable deep learning SPECT model improved obstructive CAD detection compared with traditional quantitative analysis and expert visual interpretation [3,4]. However, most existing CAD-oriented MPI studies rely on proprietary stress and rest datasets with diagnostic labels, which limits public benchmarking, reproducibility, and direct comparison across methods.

A major barrier to reproducible deep learning research in MPI is the limited availability of public datasets with usable imaging data and annotations. The PhysioNet Myocardial Perfusion Scintigraphy Image Database v1.0.0 provides an important step toward addressing this limitation. The dataset contains myocardial perfusion scintigraphy studies from 83 unique patients acquired using a CZT-based gamma camera with a rest-only ^99m^Tc-MIBI protocol. It includes raw DICOM data and preprocessed NIfTI volumes with myocardium segmentation masks where available [5,6]. Because this public release provides anatomical masks rather than fully curated CAD outcome labels, it is more suitable for segmentation-focused learning and anatomy-guided representation modeling than for direct supervised CAD classification.

This distinction is methodologically important. Many recent MPI-based CAD models assume the availability of paired stress and rest imaging, structured clinical variables, and diagnostic labels. The current public PhysioNet release does not provide these types of supervision. Therefore, a model designed for this dataset should be aligned with the available data: volumetric MPI images and corresponding myocardium masks. In this context, myocardium segmentation is a necessary first step toward reproducible MPI analysis because it supports myocardial region localization, quantitative uptake assessment, and future downstream CAD-oriented modeling when larger annotated datasets become available [5,6,7].

In this study, we propose CardioProto-SegNet, an image-only 3D anatomy-directed deep learning framework for myocardium segmentation from public myocardial perfusion scintigraphy data. The model is implemented as a 3D U-Net-like residual encoder–decoder network enhanced with squeeze-and-excitation channel recalibration and compact prototype-memory refinement at the bottleneck. Unlike prior CAD classification models that depend on proprietary diagnostic datasets or structured clinical variables, the proposed framework is specifically designed for the available PhysioNet supervision and focuses on reproducible myocardium segmentation from DICOM MPI volumes.

The main contributions of this study are as follows:

- We formulate the PhysioNet myocardial perfusion scintigraphy dataset as a reproducible public benchmark for 3D myocardium segmentation rather than as a supervised CAD diagnostic dataset.
- We introduce CardioProto-SegNet, an image-only 3D U-Net-like residual encoder–decoder architecture with squeeze-and-excitation recalibration and compact prototype-memory refinement for anatomy-aware myocardium segmentation.
- We clarify that no structured clinical variables are used in the current experiments; therefore, all reported results correspond to image-only segmentation performance.
- We provide a reproducible evaluation protocol including holdout testing, five-fold cross-validation, ablation analysis, boundary-metric evaluation, exploratory uptake-heterogeneity subgroup analysis, and comparison with common 3D segmentation baselines.

## 2. Literature Review

In recent years, deep learning has been increasingly used for Coronary Artery Disease (CAD) diagnosis, prognostic modeling, polar classification, attenuation correction, and workflow optimization of SPECT-MPI. Initial landmark studies showed that learned image representations can predict obstructive CAD better than traditional quantitative data, such as total perfusion deficit, based on MPS [8,9]. This has been further pursued in subsequent studies using graph-convolutional networks, CNN-based polar-map classification, transfer learning, and conventional machine-learning classifiers, demonstrating that compact polar-map representations can be used for automatic CAD-related classification but can potentially lose out on volumetric and anatomical information present in the original 3D image space [10,11,12,13]. More recent methods include multimodal and explainable learning, which integrate MPI images with clinical variables, radiomic features, natural language information, and attention-based fusion mechanisms, moving beyond the exclusive focus on image classification [14,15,16,17]. With respect to indirect diagnosis, deep learning models have also been applied to MPI prognosis, image-domain attenuation correction, CT-free correction, reconstruction, and preprocessing assistance in cardiac SPECT [17,18,19,20], highlighting the potential of deep learning in various stages of MPI analysis. Public and reproducible SPECT-MPI research is also starting to appear, with articles demonstrating classification feasibility for anonymized public datasets using CNN [21] and systematic reviews highlighting the potential of deep learning in diagnosis and prognosis and the ongoing need for reproducibility, external validation, and clinically viable workflows [22].

Automated myocardium segmentation is an essential step in myocardial perfusion scintigraphy and SPECT-MPI analysis because it defines the myocardial region of interest (ROI) used for uptake quantification, functional assessment, polar-map generation, perfusion defect estimation, and subsequent computer-aided interpretation. Unlike image-level CAD classification, segmentation directly addresses anatomical localization and ensures that downstream quantitative measurements are extracted from the appropriate myocardial region. This is especially relevant in SPECT-MPI, where myocardial borders may be affected by low spatial resolution, partial-volume effects, extracardiac activity, variable ventricular geometry, heterogeneous uptake, and low contrast. Therefore, accurate myocardium segmentation is a clinically and technically important step before developing downstream CAD-related models.

Traditional segmentation approaches for myocardial perfusion SPECT were primarily based on image-processing techniques such as thresholding, region growing, deformable contours, and geometric or shape constraints. Soneson et al. [23] presented an improved automatic segmentation technique for the left ventricle in myocardial perfusion SPECT and highlighted the importance of left ventricular mass segmentation for calculating perfusion defect size as a percentage of the left ventricle. Classical methods are computationally efficient and interpretable, but they may be sensitive to weak boundaries, abnormal uptake patterns, extracardiac activity, and anatomical variability. These limitations have motivated the development of learning-based techniques that can derive more expressive volumetric representations from SPECT-MPI data.

In recent years, deep learning techniques have been increasingly studied for myocardial and ventricular segmentation in SPECT-MPI. Kikuchi et al. [24] investigated automatic myocardial region extraction from transaxial SPECT images to minimize the effect of extramyocardial activity, demonstrating the importance of myocardial ROI localization for suppressing noncardiac uptake interference. Zhu et al. [25] introduced a deep learning technique using shape priors for left ventricular segmentation in myocardial perfusion SPECT images. Their method combined a 3D V-Net with shape-prior and deformation constraints to improve segmentation of endocardial, myocardial, and epicardial contours. This study shows that anatomical constraints can enhance the physiological plausibility of deep segmentation models.

Myocardial perfusion segmentation tasks have also been addressed using U-Net- and V-Net-based architectures. Zhao et al. [26] proposed a spatial–temporal V-Net with ConvLSTM skip pathways for automatic segmentation and quantification of the right ventricle in gated myocardial perfusion SPECT images, highlighting the benefits of volumetric and temporal feature learning for nuclear cardiology segmentation. Alenezi et al. [27] demonstrated the applicability of a U-Net deep learning model for SPECT-MPI segmentation, further supporting the utility of encoder–decoder architectures in myocardial perfusion image-processing pipelines. More recently, Huang et al. [28] proposed FedDA-TSformer, a federated domain-adaptation approach using a vision TimeSformer for left-ventricular segmentation in multicenter gated myocardial perfusion SPECT. This study demonstrates the need for generalization and domain adaptation in the presence of institutional, scanner, and acquisition-protocol differences.

As shown in the reviewed literature, automated segmentation in myocardial perfusion SPECT has evolved from traditional image-processing techniques to deep volumetric and domain-adaptive learning strategies. However, several challenges remain, including the small size of public datasets, domain shift, anatomical variability, heterogeneous uptake patterns, and differences in anatomical targets across studies. Many previous studies used private datasets, gated acquisitions, or task-specific annotations, making direct comparison and reproducibility difficult. In contrast, the present study uses the publicly available PhysioNet myocardial perfusion scintigraphy dataset and addresses image-only 3D myocardium segmentation using the available DICOM volumes and myocardium masks.

This literature also motivates anatomy-aware representation learning for SPECT-MPI segmentation. Myocardium segmentation requires both voxel-wise discrimination between myocardium and background and slice-wise and subject-wise anatomical consistency. Shape-prior methods address this issue by imposing constraints on plausible ventricular geometry, whereas attention- and transformer-based methods improve contextual feature modeling. Similarly, prototype-memory refinement can encourage bottleneck features to align with recurring myocardial appearance patterns. Motivated by these factors, CardioProto-SegNet combines a 3D U-Net-like residual encoder–decoder backbone, squeeze-and-excitation channel recalibration, and lightweight prototype-memory refinement into a reproducible segmentation network for public SPECT-MPI data.

Table 1 summarizes segmentation-oriented studies closely related to the present work. These studies are directly relevant to myocardial or ventricular localization, contour extraction, and segmentation-based quantitative analysis. The present study builds on previous work by providing a reproducible public dataset evaluation using the PhysioNet myocardial perfusion scintigraphy dataset, focusing on image-only myocardium segmentation, boundary metrics, ablation analysis, and comparison with common 3D segmentation baselines.

## 3. Proposed Model

**Figure 1:**
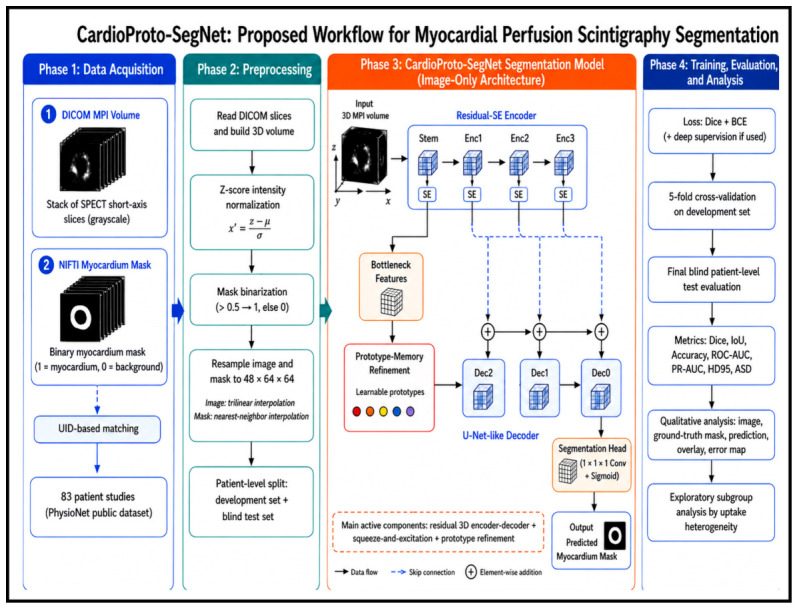
Proposed CardioProto-SegNe model.

CardioProto-SegNet, as shown in Figure 1, is designed as a three-dimensional U-Net-like residual encoder–decoder network for myocardium segmentation from myocardial perfusion scintigraphy volumes. The active components of the proposed architecture include residual 3D convolutional feature encoding, squeeze-and-excitation (SE) channel recalibration, skip-connected decoding, and compact prototype-memory refinement at the bottleneck level. Since the public PhysioNet dataset does not provide structured clinical variables, the clinical encoder and clinical-gated fusion branch were removed from the current model description. Therefore, all reported results in this study correspond to image-only myocardium segmentation.

The model receives a preprocessed 3D myocardial perfusion imaging (MPI) volume as input and produces a binary myocardium probability map as output. Let the input MPI volume be denoted by $X\in R^{D\times H\times W}$, where *D*, *H*, and *W* represent the depth, height, and width of the volumetric image, respectively. The corresponding binary myocardium mask is denoted by $Y\in {\left\{0,1\right\}}^{D\times H\times W}$. After preprocessing, the network learns a nonlinear mapping $f_{\theta }:X\to \hat{Y}$, where $\hat{Y}$ represents the predicted myocardium probability map and θ denotes the trainable parameters of the model.

### 3.1. Phase 1: Data Acquisition

The dataset can be formally represented as $D={\left\{\left(X_{i},Y_{i}\right)\right\}}_{i=1}^{N}$, where $X_{i}$ denotes the *i*-th myocardial perfusion imaging volume and $Y_{i}$ denotes its corresponding binary myocardium mask. Since the current public dataset does not provide structured clinical variables, only paired image–mask samples are used in the present experiments. This image-only formulation ensures that the training protocol is consistent with the available supervision and avoids introducing inactive or unavailable model inputs.

Algorithm 1 summarizes the data acquisition and pairing procedure. First, all myocardium mask files are scanned from the NIfTI mask directory. For each mask file, the study UID is extracted from the filename and used to identify the corresponding DICOM MPI volume. If a matching DICOM file is found, the DICOM volume and its corresponding NIfTI myocardium mask are loaded and appended as a paired training sample.
**Algorithm 1** Data acquisition and pairing**Require:** DICOM directory $D_{dcm}$, NIfTI mask directory $D_{mask}$ **Ensure:** Paired dataset $D=\{\left(X_{i},Y_{i}\right)\}$  1: Scan all myocardium mask files in $D_{mask}$.  2: **for** each mask file $Y_{i}$ **do**  3:     Extract the study UID from the filename.  4:     Search for the DICOM file $X_{i}$ with the same UID in $D_{dcm}$.  5:     **if** a matching DICOM file exists **then**  6:         Load $X_{i}$ as a DICOM MPI volume.  7:         Load $Y_{i}$ as the corresponding NIfTI myocardium mask.  8:         Append $\left(X_{i},Y_{i}\right)$ to D.  9:     **end if**  10: **end for**  11: **return** D.

### 3.2. Phase 2: Preprocessing

The preprocessing stage as shown in Algorithm 2 converts the raw DICOM images and NIfTI masks into standardized volumetric inputs suitable for 3D neural network training. The DICOM slices are first loaded and stacked into a 3D image volume. Then, intensity normalization is applied to reduce acquisition-related scale variability. Finally, both the image volume and the corresponding myocardium mask are resampled to a fixed target resolution so that all samples can be processed using a shared 3D network architecture.

The original DICOM/NIfTI studies were not assumed to have identical matrix dimensions or voxel spacing across all cases. Therefore, all image volumes and masks were standardized before model training. Image volumes were resampled to a common target grid of 48×64×64 voxels using trilinear interpolation, whereas binary myocardium masks were resampled using nearest-neighbor interpolation to preserve label integrity. This standardization step ensured a consistent input size for the 3D network and reduced memory inconsistency across training batches. Since resampling may affect fine boundary details, both overlap-based metrics and boundary-based metrics were interpreted with this preprocessing step taken into consideration.

The target resolution of 48×64×64 was selected to preserve the global 3D myocardial shape while controlling GPU memory consumption during volumetric training. Higher spatial resolutions increased memory demand and reduced the feasible batch size, whereas lower resolutions risked excessive loss of myocardial boundary detail. Therefore, this resolution was used as a practical compromise between anatomical preservation and computational feasibility.

Let the raw image volume be *X*. Its normalized form $\tilde{X}$ is computed using z-score normalization as follows:

(1) $$\tilde{X}=\frac{X-\mu (X)}{\sigma (X)+\epsilon },$$

where μ(X) and σ(X) denote the mean and standard deviation of the image volume, respectively, and ϵ is a small constant used for numerical stability. The resampling operation is expressed as follows:

(2) $$X^{*}=R_{img}\left(\tilde{X},D_{t},H_{t},W_{t}\right),$$

(3) $$Y^{*}=R_{mask}\left(Y,D_{t},H_{t},W_{t}\right),$$

where $\left(D_{t},H_{t},W_{t}\right)$ is the target 3D resolution, $R_{img}$ denotes trilinear interpolation for the image volume, and $R_{mask}$ denotes nearest-neighbor interpolation for the binary mask.
**Algorithm 2** Preprocessing**Require:** Paired sample (X,Y) **Ensure:** Standardized sample $\left(X^{*},Y^{*}\right)$  1: Read DICOM slices and stack them into a 3D volume *X*.  2: Compute the mean μ(X) and standard deviation σ(X).  3: Normalize the image volume using z-score normalization:$$\tilde{X}=\frac{X-\mu (X)}{\sigma (X)+\epsilon }.$$  4: Resample $\tilde{X}$ to the target size $\left(D_{t},H_{t},W_{t}\right)$ using trilinear interpolation.  5: Resample *Y* to the target size $\left(D_{t},H_{t},W_{t}\right)$ using nearest-neighbor interpolation.  6: **return** $\left(X^{*},Y^{*}\right)$.

### 3.3. Phase 3: CardioProto-SegNet Segmentation Model

The segmentation model is the main component of the proposed framework. It receives the preprocessed 3D MPI volume and produces a myocardium probability map through three major stages: residual-SE 3D image encoding, prototype-memory refinement at the bottleneck, and U-Net-like skip-connected decoding. Because structured clinical variables are not available in the current public dataset, the architecture is deliberately formulated as an image-only myocardium segmentation model.

#### 3.3.1. Residual-SE 3D Image Encoder

The image encoder extracts multiscale volumetric features from the MPI volume. It consists of residual 3D convolutional blocks and SE recalibration modules. Given the standardized input volume $X^{*}$, the encoder generates multiscale feature maps $F_{0}$, $F_{1}$, $F_{2}$, and $F_{b}$, where shallow features preserve spatial details and deeper features capture more abstract anatomical and perfusion-related patterns.

A residual 3D block computes the output feature representation as follows:

(4) $$F_{l+1}=\phi \left(H\left(F_{l}\right)+S\left(F_{l}\right)\right),$$

where $F_{l}$ denotes the input feature map at level *l*, H(·) is the residual convolutional transformation, S(·) is the identity or projection shortcut, and ϕ(·) is the nonlinear activation function. This residual formulation improves feature propagation and reduces gradient degradation during volumetric feature learning.

The SE recalibration module adaptively reweights channel responses. For a feature map *F*, the channel descriptor $z_{c}$ is computed by global average pooling:

(5) $$z_{c}=\frac{1}{DHW}\sum_{d=1}^{D}\sum_{h=1}^{H}\sum_{w=1}^{W}F_{c,d,h,w}.$$

The channel recalibration vector is then obtained as follows:

(6) $$s=\sigma \left(W_{2}\delta \left(W_{1}z\right)\right),$$

and the recalibrated feature map is computed as follows:

(7) $${\hat{F}}_{c}=s_{c}\cdot F_{c},$$

where δ denotes the nonlinear activation function, σ is the sigmoid function, and $W_{1}$ and $W_{2}$ are learnable parameters. The role of SE recalibration is to emphasize informative myocardial feature channels while suppressing less relevant responses.

#### 3.3.2. Prototype-Memory Refinement

To encourage compact anatomy-aware bottleneck representations, the model uses a lightweight prototype-memory refinement module. In the current implementation, the prototype memory contains K=4 learnable prototypes, denoted by $P=\{p_{1},p_{2},\ldots ,p_{K}\}$, where each prototype $p_{k}\in R^{p}$ has the same dimensionality as the projected bottleneck embedding.

The bottleneck feature map $F_{b}$ is first globally pooled to obtain a query vector:

(8) $$q=\mathrm{GAP}(F_{b}),$$

where GAP(·) denotes global average pooling. The attention weight assigned to the *k*-th prototype is computed as follows:

(9) $$a_{k}=\frac{\exp\left(q^{T}p_{k}/\sqrt{p}\right)}{\sum_{j=1}^{K}\exp\left(q^{T}p_{j}/\sqrt{p}\right)}.$$

The prototype-enhanced representation is then calculated as follows:

(10) $$r=\sum_{k=1}^{K}a_{k}p_{k}.$$

The refined bottleneck representation is obtained as follows:

(11) $$F_{ref}=F_{b}+\mathrm{Broadcast}\left(r\right).$$

The prototype-memory module is used as a lightweight anatomical refinement mechanism rather than as the primary source of model performance. Its purpose is to encourage recurring myocardial appearance patterns at the bottleneck while keeping the architecture compact for the small public dataset.

#### 3.3.3. Skip-Connected Decoder and Segmentation Head

As shown in Algorithm 3, the decoder reconstructs the myocardium probability map from the refined bottleneck representation. It follows a U-Net-like design with three decoder stages, Dec2, Dec1, and Dec0. At each stage, the decoder upsamples the feature representation and combines it with the corresponding encoder feature through skip connections. These skip connections help recover spatial details that may be lost during downsampling and improve localization of the myocardial boundary.

The final segmentation head consists of a 1×1×1 convolution followed by sigmoid activation. The sigmoid function converts the final logits into voxel-wise probabilities, producing the predicted myocardium probability map $\hat{Y}$. During inference, the probability map is thresholded to obtain the final binary myocardium mask.
**Algorithm 3** CardioProto-SegNet segmentation modeling**Require:** Preprocessed image volume $X^{*}$ Ensure: Predicted myocardium probability map $\hat{Y}$  1: Encode $X^{*}$ using the residual-SE 3D encoder.  2: Generate multiscale feature maps $\left\{F_{0},F_{1},F_{2},F_{b}\right\}$.  3: Apply global average pooling to $F_{b}$ to obtain query vector *q*.  4: Compute attention weights between *q* and the learnable prototype set ${\left\{p_{k}\right\}}_{k=1}^{K}$.  5: Aggregate the prototype response $r=\sum_{k=1}^{K}a_{k}p_{k}$.  6: Broadcast *r* and add it to $F_{b}$ to obtain $F_{ref}$.  7: Decode $F_{ref}$ using skip-connected decoder stages Dec2, Dec1, and Dec0.  8: Apply a 1×1×1 convolution and sigmoid activation to obtain $\hat{Y}$.  9: **return** $\hat{Y}$. 

### 3.4. Architecture Configuration

Table 2 presents the detailed configuration of the CardioProto-SegNet architecture used in the current experiments. The model receives a one-channel 3D MPI volume with an input size of 1×48×64×64 and processes it through a 3D convolutional stem followed by residual-SE feature extraction. The encoder consists of three downsampling stages, Enc1, Enc2, and Enc3, with channel widths of 48, 96, and 192, respectively, using a base width of 24 channels. Residual 3D convolutional blocks are used in the encoder and bottleneck to improve feature propagation, while SE recalibration is applied after each encoder stage to adaptively emphasize informative channel responses. At the bottleneck, a compact prototype-memory module containing K=4 learnable prototypes refines the residual-SE representation. The decoder includes Dec2, Dec1, and Dec0 stages, where encoder features are connected to the corresponding decoder features through skip connections. The final segmentation head consists of a 1×1×1 convolution followed by sigmoid activation to generate the binary myocardium probability map. The model was optimized using a combined Dice loss and binary cross-entropy loss, with optional deep supervision, and trained using the AdamW optimizer with a learning rate of $8\times 10^{-4}$ and weight decay of $1\times 10^{-4}$. The training setting used a batch size of 16, a maximum of 50 epochs, and early stopping with a patience of 10 epochs.

### 3.5. Phase 4: Evaluation Metrics

CardioProto-SegNet’s segmentation performance was assessed using overlap-based, voxel-level, probability-based, and boundary-based metrics. This multi-metric evaluation strategy was used because medical image segmentation quality cannot be fully defined using a single metric, especially for volumetric tasks with strong foreground–background imbalance. The Dice similarity coefficient and Jaccard/IoU are commonly used to measure spatial overlap between predicted and reference segmentations, while surface-distance metrics such as Hausdorff distance and average surface distance provide additional insight into boundary localization accuracy [29,30,31,32].

Let TP, TN, FP, and FN denote the number of true-positive, true-negative, false-positive, and false-negative voxels, respectively. The Dice similarity coefficient was used to quantify the overlap between the predicted myocardium mask and the reference mask [29,30]:

(12) $$\mathrm{Dice}=\frac{2TP}{2TP+FP+FN}.$$

The intersection over union (IoU), also known as the Jaccard index, was used as an additional overlap-based metric [31]:

(13) $$\mathrm{IoU}=\frac{TP}{TP+FP+FN}.$$

Voxel-level accuracy was calculated as follows:

(14) $$\mathrm{Accuracy}=\frac{TP+TN}{TP+TN+FP+FN}.$$

Although accuracy was reported for completeness, it was interpreted with caution because the myocardium occupies a much smaller volume than the background class in the 3D MPI volume. Therefore, Dice, IoU, PR-AUC, HD95, and ASD were considered more informative for evaluating myocardial segmentation performance under class imbalance [32,33].

ROC-AUC and PR-AUC were used to evaluate probability-based discrimination. Sensitivity, also known as recall or the true-positive rate, was defined as follows:

(15) $$\mathrm{Sensitivity}=\frac{TP}{TP+FN}.$$

Specificity was calculated as follows:

(16) $$\mathrm{Specificity}=\frac{TN}{TN+FP}.$$

The false-positive rate was determined as follows:

(17) $$\mathrm{FPR}=\frac{FP}{FP+TN}.$$

The ROC curve was created by plotting sensitivity against the false-positive rate for different probability thresholds, and ROC-AUC was calculated as the area under this curve. Precision was determined as follows:

(18) $$\mathrm{Precision}=\frac{TP}{TP+FP}.$$

The precision–recall curve was created by plotting precision against recall at different probability thresholds, and PR-AUC was computed as the area under this curve. PR-AUC was included because it is especially informative when the foreground class is small relative to the background class, as in myocardium segmentation from 3D MPI volumes [33].

Boundary performance was evaluated using the 95th percentile Hausdorff distance (HD95) and average surface distance (ASD). These metrics are commonly used to evaluate contour and surface agreement in 3D medical image segmentation [32]. Let S(P) and S(G) denote the surface voxels of the predicted mask *P* and the ground-truth mask *G*, respectively. The shortest distance between a surface point p∈S(P) and the ground-truth surface S(G) is defined as follows:

(19) $$d\left(p,S\left(G\right)\right)=\min_{g\in S(G)}∥p-g∥.$$

The bidirectional surface-distance set was calculated from the predicted surface to the ground-truth surface and from the ground-truth surface to the predicted surface. HD95 was computed as follows:

(20) $$\mathrm{HD}95={\mathrm{percentile}}_{95}\left(\{d(p,S(G))|p\in S(P)\}\cup \{d(g,S(P))|g\in S(G)\}\right).$$

The average surface distance was computed as follows:

(21) $$\mathrm{ASD}=\mathrm{mean}\left(\{d(p,S(G))|p\in S(P)\}\cup \{d(g,S(P))|g\in S(G)\}\right).$$

Lower HD95 and ASD values indicate better boundary agreement between the predicted myocardium mask and the reference annotation. Together, these metrics provide complementary information: Dice and IoU evaluate volumetric overlap, accuracy reports global voxel-level correctness, ROC-AUC and PR-AUC evaluate probabilistic foreground–background discrimination, and HD95 and ASD evaluate boundary localization accuracy.

## 4. Experimental Results and Discussion

### 4.1. Dataset

The experiments were conducted using the Myocardial Perfusion Scintigraphy Image Database v1.0.0, a publicly available PhysioNet dataset containing 83 patient studies acquired in a real clinical imaging environment. The studies were obtained using a Discovery NM 530c CZT-based gamma camera (GE Healthcare) with a rest-only ^99m^Tc-MIBI protocol. The dataset provides raw DICOM myocardial perfusion scintigraphy volumes and corresponding NIfTI myocardium segmentation masks for selected cases, making it suitable for reproducible myocardium segmentation and anatomy-aware representation learning rather than direct supervised CAD classification [5].

**Figure 2:**
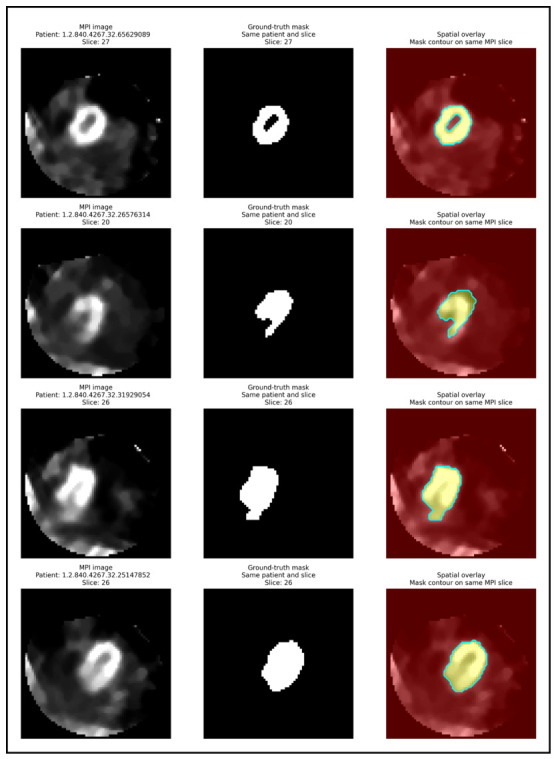
Representative myocardial perfusion scintigraphy images, ground-truth myocardium masks, and spatial overlays. Each row shows one representative patient slice from the public MPS dataset. The first column presents the grayscale MPI image, the second column shows the corresponding binary ground-truth myocardium mask, and the third column presents the spatial overlay of the mask on the same MPI slice. In the overlay panels, the red background represents the MPI slice intensity map, the yellow region indicates the myocardium mask area, and the cyan contour shows the boundary of the ground-truth myocardium mask.

Sample spatially matched examples are displayed in Figure 2. The DICOM MPI slice, the binary myocardium mask, and the overlay visualization are presented in each row for the same patient and slice index. The overlay shows the spatial overlap between the perfusion image and the annotated myocardial region and demonstrates inter-patient variation in myocardial appearance and uptake distribution.

The segmentation problem is highly imbalanced at the voxel level because myocardial voxels account for only a small fraction of the entire 3D volume, whereas background voxels dominate. In the analyzed cohort, the average proportion of myocardial foreground voxels was 1.54%, while the background proportion was 98.46%. Therefore, voxel-level accuracy was interpreted with caution and was not used as the primary criterion for segmentation performance.

A limitation of the public dataset is the absence of confirmed ischemia, infarction, or patient-level CAD outcome labels. Therefore, segmentation performance across clinically verified normal, ischemic, and infarcted subgroups could not be directly assessed in the present study. To partially address this limitation, an exploratory image-derived subgroup analysis was performed using myocardial ROI uptake statistics. This analysis was used to examine segmentation behavior in lower- and higher-uptake-heterogeneity groups and should not be interpreted as clinical disease classification.

All experiments used patient-level partitioning to improve reproducibility and reduce the risk of data leakage. Each patient study was assigned to only one data partition, ensuring that no patient appeared simultaneously in the training and validation/test sets. All splits were generated at the patient level after DICOM–mask matching. Five-fold cross-validation was used internally for validation with a fixed random seed of 42. In addition, a stricter evaluation protocol was adopted by separating a development subset from an untouched blind test subset. Five-fold cross-validation was performed only on the development subset, and the final model was evaluated once on the blind patient-level test subset.

### 4.2. Implementation Details

All myocardial perfusion volumes and corresponding masks were resampled to 48×64×64 voxels to ensure uniform volumetric inputs during training. Image volumes were normalized using z-score normalization, and binary masks were used as segmentation targets during model training. The AdamW optimizer was used with an initial learning rate of $8\times 10^{-4}$ and a weight decay of $1\times 10^{-4}$. The model was trained for a maximum of 50 epochs, with an early stopping patience of 10 epochs based on the validation Dice score. A cosine learning-rate annealing schedule was used to improve convergence stability, and mixed-precision training was applied when CUDA support was available.

The loss function combined binary cross-entropy loss and soft Dice loss with weights of 0.35 and 0.65, respectively. Optional deep supervision was applied to decoder outputs with a weight of 0.25. During training, 3D image volumes and masks were augmented on the fly using random spatial flipping. To ensure reproducibility, Python, NumPy, and PyTorch were seeded with a fixed value of 42. The implementation code is publicly available on GitHub at https://github.com/KOkab2020/3DCardioProto.git (accessed on 20 April 2026).

ROI statistics were computed for each patient in the exploratory uptake-heterogeneity analysis using the reference myocardium mask. The extracted features included mean uptake, standard deviation, coefficient of variation, percentile-based spread, and the proportion of low-uptake voxels. Patients were then stratified into lower- and higher-heterogeneity groups based on the median ROI heterogeneity score. This subgrouping was used only as an image-derived surrogate of segmentation difficulty and not as a clinical diagnosis of ischemia or infarction.

All experiments were conducted on a workstation equipped with a Tesla T4 GPU with 14.6 GB of memory, an Intel(R) Xeon(R) CPU at 2.00 GHz, and 31.4 GB of RAM. The software environment included Python 3.12.12, PyTorch 2.9.0+cu126, and CUDA 12.6. The average training time for CardioProto-SegNet was approximately 6.50 min per fold.

### 4.3. Results

**Figure 3:**
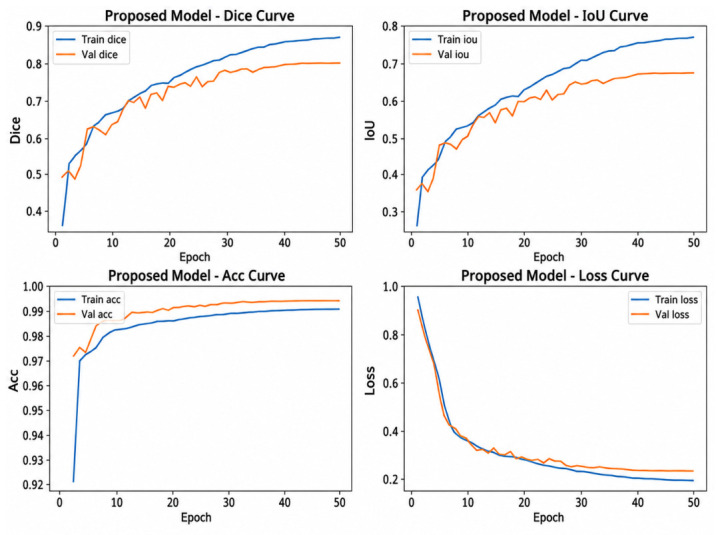
Training and validation learning curves of the proposed CardioProto-SegNet model for a representative development fold across 50 epochs.

The training and validation learning curves of CardioProto-SegNet for a representative development fold over 50 epochs are illustrated in Figure 3. The Dice and IoU curves for the training and validation sets started at relatively low values and gradually increased as the model improved its myocardium delineation accuracy. The validation Dice stabilized at approximately 0.82, while the validation IoU reached approximately 0.69 toward the end of training. This indicates stable segmentation learning in the representative fold.

The voxel-level accuracy curve increased rapidly during the early epochs and then plateaued at a high level. However, this value should be interpreted with caution because the 3D segmentation volume is dominated by background voxels. Therefore, Dice, IoU, PR-AUC, HD95, and ASD were considered more informative for evaluating myocardium segmentation quality. The training and validation loss curves both decreased gradually and remained close to each other, indicating that no severe overfitting was observed in the representative fold during training.

The five-fold cross-validation results of CardioProto-SegNet are shown in Table 3. The model showed stable overlap performance across the five folds, with Dice scores ranging from 0.7635 to 0.8504 and IoU values ranging from 0.6326 to 0.7154. Overall, the model achieved a mean Dice score of 0.8239±0.0350 and a mean IoU of 0.6870±0.0328, indicating consistent myocardium segmentation performance across patient-level folds. Because of the strong foreground–background imbalance, the mean voxel-level accuracy of 0.9943±0.0004 was interpreted as a secondary measure rather than a primary segmentation metric. The probability-based results showed good foreground–background discrimination, with a mean ROC-AUC of 0.9867±0.0047 and a mean PR-AUC of 0.8561±0.0829. The larger variation in PR-AUC reflects the sensitivity of precision–recall performance to myocardial foreground imbalance and fold composition.

HD95 and ASD were also used to evaluate boundary performance. The mean HD95 and mean ASD were 2.5181±1.0593 and 0.9261±0.5016, respectively, indicating acceptable boundary agreement between the predicted and reference myocardium masks. Fold 4 produced the lowest Dice, IoU, ROC-AUC, and PR-AUC values and also showed the highest boundary errors. This suggests that the cases in this fold were more challenging, possibly due to weaker myocardial borders, more heterogeneous uptake patterns, or increased image–mask complexity. Overall, the five-fold results demonstrate consistent myocardium segmentation performance while also highlighting the variability and difficulty of this small public MPI dataset.

**Figure 4:**
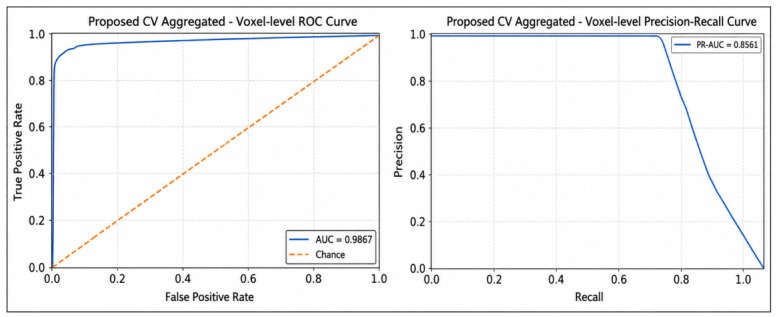
Aggregated voxel-level ROC and precision–recall curves of the proposed CardioProto-SegNet model across five-fold cross-validation.

The aggregated voxel-level ROC and precision–recall curves of the proposed CardioProto-SegNet model across the five-fold cross-validation experiment are shown in Figure 4. The ROC curve demonstrates strong foreground–background discrimination, with an AUC of 0.9867. The curve remains clearly above the diagonal chance line, indicating that the model can distinguish myocardial voxels from background voxels across a broad range of decision thresholds.

The precision–recall curve provides a complementary assessment that is particularly relevant for this task because myocardial voxels represent only a small fraction of the full 3D volume. The model achieved a high PR-AUC of 0.8561, indicating good precision–recall performance under strong foreground–background imbalance. Precision remained high over a broad recall range, suggesting that the model can retrieve a large proportion of myocardial voxels while maintaining a relatively low false-positive rate. The decline in precision at very high recall values reflects the expected trade-off: when the decision threshold is lowered to capture nearly all myocardial voxels, more background voxels are also included as false positives. Overall, these curves confirm that CardioProto-SegNet generates meaningful voxel-level probability estimates and provides effective myocardium–background separation across the cross-validation folds.

#### Exploratory Subgroup Analysis Based on Myocardial ROI Uptake Heterogeneity

Myocardial ROI uptake heterogeneity was used to perform an exploratory subgroup analysis because the public dataset does not provide confirmed ischemia or infarction labels. Patients were divided into lower- and higher-heterogeneity groups according to the median ROI heterogeneity score. This analysis was not designed to determine disease status; rather, it was performed to examine whether segmentation performance changed as myocardial uptake became more heterogeneous and potentially more difficult to delineate.

**Figure 5:**
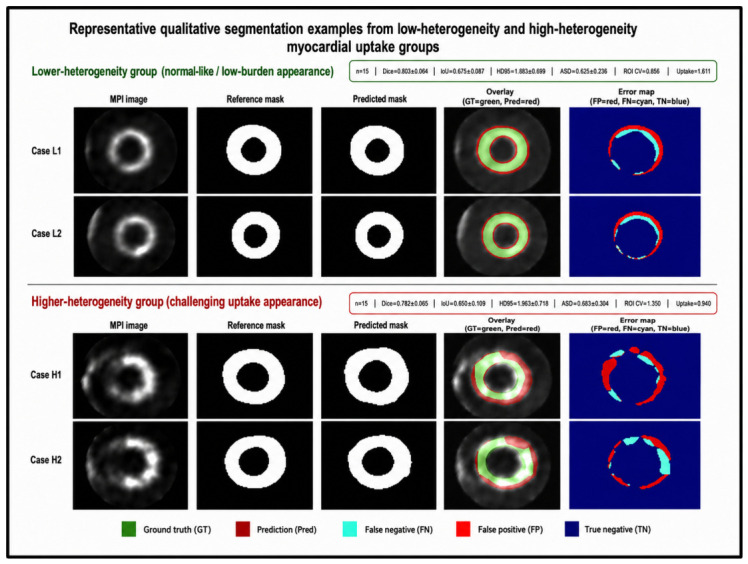
Representative qualitative segmentation examples from low-heterogeneity and high-heterogeneity myocardial uptake groups.

As shown in Table 4 and Figure 5, myocardial ROI uptake heterogeneity had an observable effect on segmentation performance. The lower-heterogeneity group showed slightly better quantitative results, with Dice of 0.8026±0.0656 and IoU of 0.6748±0.0870, compared with Dice of 0.7824±0.0853 and IoU of 0.6497±0.1092 in the higher-heterogeneity group. A similar trend was observed for boundary performance, with the lower-heterogeneity group achieving lower HD95 and ASD values, indicating better boundary localization. The ROI statistics support this interpretation: the higher-heterogeneity group had a higher ROI coefficient of variation and lower mean uptake, suggesting a more irregular tracer distribution and more challenging myocardial borders.

Figure 5 visually supports the quantitative subgroup findings. Lower-heterogeneity examples show smoother uptake patterns and closer agreement between the reference and predicted masks, whereas higher-heterogeneity examples show more visible false-positive and false-negative regions in the error maps. Since confirmed ischemia or infarction labels were unavailable, this analysis should be interpreted only as an exploratory image-derived assessment of segmentation difficulty.

**Figure 6:**
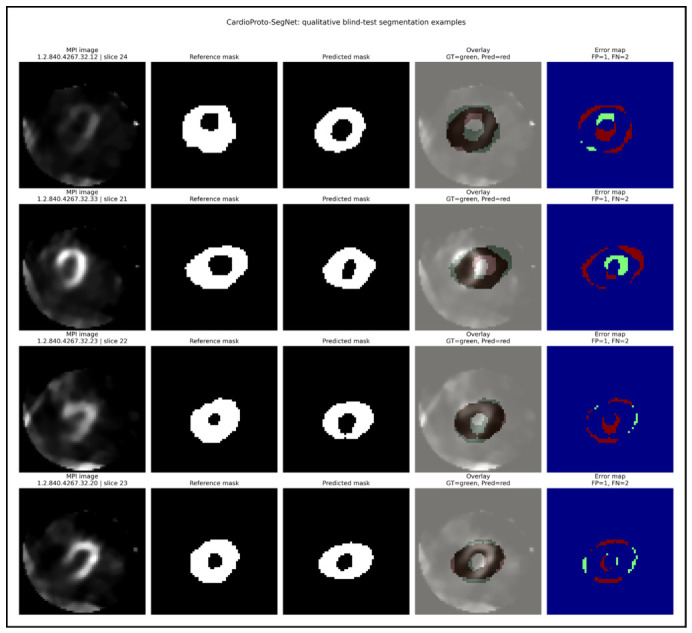
Qualitative blind-test segmentation examples of CardioProto-SegNet for myocardial perfusion scintigraphy myocardium segmentation. Each row presents one representative blind-test MPI slice with its corresponding reference mask, predicted mask, spatial overlay, and error map. The first column shows the grayscale MPI image, the second column shows the binary reference myocardium mask, and the third column shows the predicted myocardium mask generated by CardioProto-SegNet. The overlay panel compares the reference and predicted contours on the same MPI slice, where the green contour represents the ground truth and the red contour represents the predicted myocardium boundary. The error map summarizes voxel-level agreement and disagreement, where red indicates false-positive regions, cyan indicates false-negative regions, and dark blue indicates true-negative background regions. The figure illustrates the spatial correspondence between the predicted and reference myocardial regions across representative blind-test cases.

Qualitative blind-test segmentation examples of CardioProto-SegNet are shown in Figure 6. Each row represents one test case and includes the original MPI slice, reference myocardium mask, predicted mask, ground-truth and prediction overlay, and error map. The predicted masks generally follow the ring-like shape and anatomical location of the reference myocardium, as shown in the overlay panels. The error maps indicate that most false-positive and false-negative regions are concentrated around the rim of the myocardial mask rather than being widely distributed across the full image. These qualitative results complement the quantitative findings and suggest that the model can generate anatomically plausible myocardium masks, although further improvements in boundary refinement may still be beneficial.

### 4.4. Ablation Study

Table 5 presents the ablation study used to assess the contribution of the main architectural components of CardioProto-SegNet. The proposed model achieved a Dice score of 0.8239, IoU of 0.6870, accuracy of 0.9943, ROC-AUC of 0.9867, and PR-AUC of 0.8561. The largest performance reduction was observed when residual connections were removed, with Dice decreasing to 0.7448 and IoU decreasing to 0.5981. This finding indicates that residual learning plays an important role in stable volumetric feature propagation and effective myocardium segmentation.

The wider network variant achieved a Dice score of 0.7972 and an IoU of 0.6694, indicating that increasing network width alone did not improve performance over the proposed configuration. The no-prototype variant achieved a Dice score of 0.8009 and an IoU of 0.6751, showing only a modest reduction compared with the proposed model. Its PR-AUC was also comparable to that of the proposed model, suggesting that prototype-memory refinement is not the primary source of model performance but instead provides a small anatomical refinement effect. The deeper network variant achieved the best overall ablation performance, with Dice of 0.8290, IoU of 0.7096, accuracy of 0.9949, and PR-AUC of 0.8831. This suggests that additional representational depth may further improve segmentation quality for this dataset; however, additional parameter-matched comparisons would be required to separate the effect of depth from the effect of model capacity.

The ablation results also clarify the relative contribution of the prototype-memory module. Compared with the proposed model, removing the prototype-memory module reduced Dice by 0.0230 and IoU by 0.0119. The no-prototype variant also maintained a similar PR-AUC. Therefore, the prototype-memory module should not be interpreted as the main driver of performance improvement. Instead, it acts as a lightweight anatomical refinement mechanism, while the residual-SE 3D encoder–decoder backbone remains the primary component responsible for the segmentation capability of the model.

**Figure 7:**
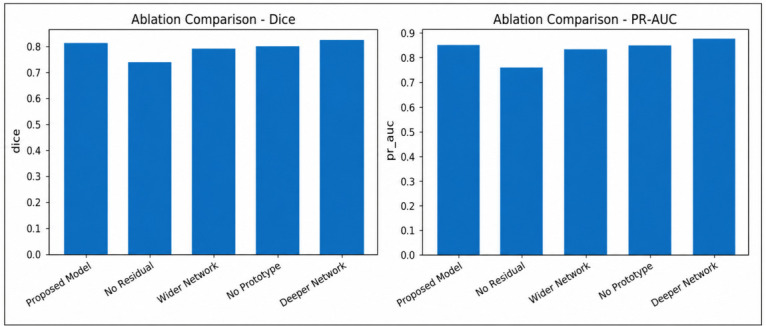
Ablation comparison of CardioProto-SegNet and its architectural variants using Dice score and PR-AUC.

Figure 7 visualizes the ablation results using Dice and PR-AUC. The no-residual variant showed the lowest performance in both metrics, confirming the importance of residual connections. The proposed, no-prototype, and wider variants remained relatively close, while the deeper variant achieved the highest values. Overall, the ablation results indicate that residual learning is essential, prototype-memory refinement provides a small but useful contribution to segmentation consistency, and the proposed model offers a design that balances performance, complexity, and reproducibility.

### 4.5. Baseline Comparison

To ensure a transparent and reproducible baseline comparison, all baseline architectures were implemented from scratch in PyTorch using the same preprocessing pipeline, input size, patient-level split, optimizer, loss function, batch size, maximum number of epochs, and early-stopping strategy used for the proposed model. No pretrained weights or external model checkpoints were used. The compared baselines included standard 3D U-Net [34], V-Net [35], Residual 3D U-Net [36], and Attention 3D U-Net [37]. These models were selected because they represent commonly used volumetric encoder–decoder segmentation architectures in medical imaging.

Table 6 summarizes the implementation details of CardioProto-SegNet and the baseline models. All models used the same input size of 1×48×64×64, four resolution levels, and a base channel width of 24. The standard 3D U-Net used double 3D convolution blocks with max-pooling and transposed-convolution upsampling. V-Net and Residual 3D U-Net incorporated residual volumetric blocks, while Attention 3D U-Net used attention-gated skip connections. CardioProto-SegNet combined residual 3D encoder–decoder blocks, SE recalibration, compact prototype memory with K=4 learnable prototypes, and deep supervision.

The proposed model contained 6,008,755 trainable parameters, which is fewer than that of the Residual 3D U-Net (6,499,969 parameters) but more than those of the standard 3D U-Net, V-Net, and Attention 3D U-Net. This comparison shows that the performance of CardioProto-SegNet cannot be explained solely by having the largest number of trainable parameters, because the Residual 3D U-Net had a larger parameter count but did not achieve better performance. Therefore, the baseline comparison supports the contribution of the proposed architectural components while keeping the evaluation transparent and controlled.

Table 7 compares the segmentation performance of CardioProto-SegNet with the four baseline models. The proposed model achieved the highest Dice score, IoU, pixel accuracy, and PR-AUC, indicating better overlap accuracy and stronger foreground precision–recall behavior. Although V-Net achieved the highest ROC-AUC, its Dice, IoU, and PR-AUC were lower than those of the proposed model. This suggests that high global voxel separability does not necessarily translate into better myocardial mask overlap under strong class imbalance.

**Figure 8:**
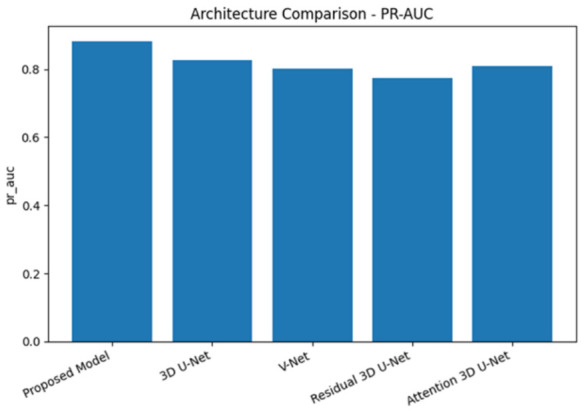
PR-AUC architecture comparison.

Figure 8 shows the PR-AUC comparison between CardioProto-SegNet and the baseline architectures. PR-AUC is particularly useful for this dataset because myocardial voxels represent only a small fraction of the full volume. The proposed model achieved the highest PR-AUC, indicating a better balance between precision and recall in identifying myocardial voxels across different probability thresholds. This supports the practical benefit of the proposed segmentation-focused design under foreground–background imbalance.

### 4.6. Statistical Analysis

Myocardial voxels occupy only a small fraction of the full volumetric image; therefore, voxel-level accuracy may be artificially high because of the dominant background class. For this reason, Dice, IoU, PR-AUC, HD95, and ASD were used as the main evaluation criteria in the statistical analysis, while accuracy was treated as a secondary metric. HD95 and ASD were included because small contour errors may be clinically relevant for downstream ROI-based uptake quantification in myocardium segmentation.

The paired statistical comparison between CardioProto-SegNet and the baseline 3D segmentation models on the blind patient-level test set is presented in Table 8. CardioProto-SegNet consistently outperformed the baseline models in terms of Dice and IoU. The average Dice improvements ranged from 0.0380 against 3D U-Net to 0.0549 against Attention 3D U-Net, while the average IoU improvements ranged from 0.0360 against 3D U-Net to 0.0558 against Attention 3D U-Net. These improvements remained statistically significant after Holm–Bonferroni correction. Small but statistically significant improvements were also observed for pixel accuracy; however, accuracy was treated as a secondary metric because the segmentation volume contains a large proportion of background voxels.

Boundary-based metrics require a different interpretation because lower HD95 and ASD values indicate better boundary agreement. Therefore, negative mean paired differences indicate lower boundary errors for CardioProto-SegNet compared with the corresponding baseline. The proposed model significantly improved HD95 compared with V-Net and Attention 3D U-Net and significantly improved ASD compared with V-Net. However, the reductions in HD95 and ASD compared with 3D U-Net were not statistically significant after Holm–Bonferroni correction. Compared with Residual 3D U-Net, CardioProto-SegNet achieved better Dice, IoU, and pixel accuracy, whereas Residual 3D U-Net achieved lower HD95 and ASD values. Overall, the paired analysis supports the effectiveness of CardioProto-SegNet for overlap-based myocardium segmentation while also indicating that boundary refinement remains an important direction for future improvement.

## 5. Discussion

### 5.1. Overall Segmentation Performance

The experimental results demonstrate that CardioProto-SegNet provides a reliable image-only framework for myocardium segmentation from publicly available myocardial perfusion scintigraphy data. Despite the limited number of patient studies and the heterogeneity commonly observed in clinical SPECT-MPI acquisitions, the model was able to learn meaningful myocardial representations. This was reflected in the holdout test Dice score of 0.7402 and further supported by the five-fold cross-validation results, in which the model achieved a mean Dice score of 0.8239 and a mean IoU of 0.6870. These findings indicate that the proposed framework can provide consistent myocardium segmentation across patient-level partitions and is not dependent on a single favorable train–validation split.

### 5.2. Interpretation of Overlap, Probability, and Boundary Metrics

The use of overlap-based, probability-based, and boundary-based metrics provides a more complete understanding of model behavior. The five-fold cross-validation results showed strong foreground–background discrimination, with a mean ROC-AUC of 0.9867 and a mean PR-AUC of 0.8561. However, voxel-level accuracy should be interpreted cautiously because background voxels dominate the 3D segmentation volume. In this context, Dice, IoU, PR-AUC, HD95, and ASD are more informative for evaluating myocardium segmentation quality. PR-AUC is particularly important because it reflects the precision–recall trade-off under strong foreground–background imbalance, while HD95 and ASD provide complementary information about myocardial boundary localization.

The probability-based results also suggest that the network can produce meaningful voxel-level confidence estimates rather than only binary masks after thresholding. This is important for future confidence-sensitive segmentation analysis, uncertainty-aware interpretation, and downstream pipelines in which the reliability of predicted myocardial boundaries may influence ROI-based quantification.

### 5.3. Fold-Wise Variability and Dataset Difficulty

The fold-wise results highlight the difficulty of the dataset. Fold 4 showed the lowest Dice, IoU, ROC-AUC, and PR-AUC values and the highest HD95 and ASD values. This suggests that this fold may have contained more challenging cases with heterogeneous tracer uptake, weaker myocardial borders, lower contrast, or greater anatomical variability. In a small public dataset, the composition of each fold can strongly affect validation performance. Therefore, the lower performance in Fold 4 should be interpreted as evidence of dataset heterogeneity rather than simply as model failure.

This finding reinforces the importance of patient-level validation, blind testing, and future external multicenter evaluation. It also emphasizes that segmentation models for myocardial perfusion scintigraphy should be assessed using multiple complementary metrics rather than relying only on accuracy or a single overlap score.

### 5.4. Contribution of Architectural Components

The ablation analysis clarified the relative importance of the main architectural components. Removing residual connections caused the largest performance reduction, with Dice decreasing from 0.8239 to 0.7448 and IoU decreasing from 0.6870 to 0.5981. This indicates that residual learning is essential for stable volumetric feature propagation and effective representation learning in the 3D encoder–decoder backbone. Residual connections likely improve gradient flow and reduce representational degradation across convolutional stages, which is especially important in 3D segmentation networks trained on small medical datasets.

The wider network variant remained competitive but did not outperform the proposed configuration, indicating that increasing channel width alone is not sufficient to improve segmentation performance. In contrast, the deeper network variant achieved the best ablation performance, with Dice of 0.8290 and IoU of 0.7096. This suggests that additional representational depth may help capture more complex anatomical and uptake-related patterns in MPI volumes. However, further parameter-matched comparisons are required to separate the effect of network depth from the effect of model capacity.

### 5.5. Role of Prototype-Memory Refinement

The prototype-memory component should be interpreted as a lightweight anatomical refinement mechanism rather than the main driver of performance improvement. Removing the prototype module caused only a modest reduction in overlap metrics, with Dice decreasing from 0.8239 to 0.8009 and IoU decreasing from 0.6870 to 0.6751. The no-prototype variant also maintained a comparable PR-AUC.

These findings suggest that the core segmentation capability of the model is mainly provided by the residual-SE 3D encoder–decoder backbone, while prototype-memory refinement contributes by encouraging compact and anatomically consistent bottleneck representations. Conceptually, the learnable prototypes act as global feature templates that represent recurring myocardial appearance patterns across subjects. This may help improve anatomical consistency, especially in cases with weak boundaries, heterogeneous uptake, or image noise. Nevertheless, its contribution remains complementary rather than dominant.

### 5.6. Baseline Comparison and Model Capacity

The baseline comparison was designed to ensure transparency and avoid overinterpreting performance differences. All baseline models were implemented from scratch in PyTorch using the same preprocessing pipeline, input resolution, patient-level split, optimizer, loss function, batch size, maximum number of epochs, and early-stopping strategy. No external checkpoints or pretrained weights were used. This controlled implementation allows the comparison to focus on architectural differences rather than differences in training protocol or external pretraining.

Under the same experimental protocol, CardioProto-SegNet achieved the best Dice, IoU, pixel accuracy, and PR-AUC among the evaluated baselines. Although V-Net achieved the highest ROC-AUC, its Dice, IoU, and PR-AUC were lower than those of the proposed model. This indicates that high global voxel separability does not necessarily translate into better myocardial mask overlap under strong foreground–background imbalance.

The parameter-count analysis further supports this interpretation. CardioProto-SegNet contained approximately 6.01 million trainable parameters, whereas Residual 3D U-Net contained approximately 6.50 million trainable parameters. Although the residual baseline had more trainable parameters, it did not achieve better Dice, IoU, pixel accuracy, or PR-AUC. Therefore, the observed performance cannot be explained solely by model size. Instead, the combination of residual 3D encoding, squeeze-and-excitation recalibration, skip-connected decoding, and compact prototype refinement appears to provide a favorable balance between representation capacity and segmentation performance.

### 5.7. Statistical Comparison and Boundary Refinement

The paired statistical analysis further supports the baseline comparison while also clarifying the limitations of the proposed model. For Dice, IoU, and pixel accuracy, positive paired differences favored CardioProto-SegNet because higher values indicate better performance. The proposed model showed statistically significant improvements over all evaluated baselines in Dice and IoU after Holm–Bonferroni correction, indicating stronger volumetric overlap performance. Pixel accuracy also showed small but significant improvements, although it was interpreted as a secondary metric because of the dominance of background voxels.

For HD95 and ASD, negative paired differences indicate better performance of CardioProto-SegNet because lower boundary distance values represent more accurate surface agreement. The proposed model significantly reduced HD95 compared with V-Net and Attention 3D U-Net and significantly reduced ASD compared with V-Net. However, the reductions in HD95 and ASD compared with 3D U-Net were not statistically significant after Holm–Bonferroni correction. In comparison with Residual 3D U-Net, CardioProto-SegNet achieved better Dice, IoU, and pixel accuracy, but Residual 3D U-Net showed lower HD95 and ASD values. This indicates that although CardioProto-SegNet provided stronger volumetric overlap and foreground discrimination, further refinement of boundary localization remains an important direction for future work.

### 5.8. Methodological and Clinical Relevance

The current study supports the suitability of a segmentation-focused formulation for the available public PhysioNet dataset. The dataset provides rest-only MPI studies with myocardium masks but does not include fully curated coronary artery disease outcome labels or structured clinical variables. Therefore, it is not appropriate to benchmark the present model as a supervised CAD classifier. Instead, the value of this work lies in providing a reproducible, anatomy-aware segmentation framework for public myocardial perfusion scintigraphy data.

Clinically, accurate myocardium segmentation can support several downstream MPI analysis tasks. Reliable myocardial localization may improve ROI-based uptake quantification, polar-map construction, perfusion-defect estimation, radiomic feature extraction, and future CAD-oriented modeling when clinically annotated datasets become available. Thus, the contribution of this work is not limited to segmentation accuracy alone; it may also provide a useful front end for future computer-aided nuclear cardiology workflows.

### 5.9. Limitations and Future Work

Several limitations should be acknowledged. First, the dataset is relatively small, which limits the diversity of myocardial anatomy, uptake patterns, scanner conditions, and reconstruction settings available for training and testing. Second, all experiments were performed using a single public dataset, so external generalizability to other institutions, scanners, tracers, and acquisition protocols has not yet been established. Third, although the deeper variant achieved the best ablation performance, the current study did not exhaustively optimize all possible capacity–depth trade-offs. Further parameter-matched and depth-matched experiments are needed to separate the effects of architecture, model capacity, and training configuration.

Another important limitation is the lack of confirmed ischemia and infarction labels. This prevents direct evaluation of segmentation performance across clinically verified normal, ischemic, and infarcted myocardium. To partially address this limitation, an exploratory subgroup analysis was performed using myocardial ROI uptake heterogeneity as an image-derived surrogate of segmentation difficulty. The results suggested that cases with more heterogeneous uptake may be more challenging for the model. However, this subgrouping should not be interpreted as clinical disease classification. Future work should validate the model using larger multicenter datasets with expert-confirmed ischemia, infarction, scar burden, stress and rest imaging, scanner diversity, and clinical outcome labels.

## 6. Conclusions

This paper introduced CardioProto-SegNet, an image-only 3D anatomy-directed deep learning framework for myocardium segmentation from publicly available myocardial perfusion scintigraphy data. The model was developed based on the supervision available in the PhysioNet Myocardial Perfusion Scintigraphy Image Database and uses a residual 3D encoder–decoder architecture with squeeze-and-excitation recalibration, skip-connected decoding, and compact prototype-memory refinement. Since the public dataset does not include structured clinical variables or confirmed CAD outcome labels, the proposed framework was evaluated as a myocardium segmentation model rather than as a diagnostic CAD classification system.

The experimental results demonstrated consistent myocardium segmentation performance, with a Dice score of 0.7402 on the holdout test set and a mean Dice score of 0.8239 across five-fold cross-validation. The model also achieved a mean IoU of 0.6870, a mean ROC-AUC of 0.9867, and a mean PR-AUC of 0.8561, indicating effective overlap performance and strong voxel-level foreground–background discrimination. The ablation analysis showed that residual learning was the most important architectural component, while prototype-memory refinement provided a modest anatomical refinement benefit. The baseline comparison and the paired statistical analysis showed that the proposed model achieved better Dice, IoU, pixel accuracy, and PR-AUC than several standard 3D segmentation baselines under the same experimental protocol. However, boundary distance results also showed that further improvement is needed for fine surface localization, particularly when compared with the Residual 3D U-Net baseline.

Although these findings are promising, several limitations remain. The dataset is relatively small, based on rest-only acquisitions, and lacks confirmed ischemia, infarction, or patient-level CAD diagnostic labels. Therefore, the present study cannot directly assess segmentation performance in clinically verified normal, ischemic, and infarcted myocardium. Future work should validate the framework using larger multicenter datasets with stress and rest imaging, expert-confirmed disease labels, scanner diversity, and clinical outcome information. Additional studies should also investigate parameter-matched and depth-matched architectures, improved boundary refinement modules, and external validation across different acquisition protocols.

Overall, CardioProto-SegNet provides a reproducible public dataset benchmark for anatomy-aware myocardium segmentation in myocardial perfusion scintigraphy. The framework may serve as a foundation for future downstream MPI analysis, including ROI-based uptake quantification, polar-map generation, radiomic feature extraction, transfer learning, and CAD-oriented decision support models when larger clinically annotated datasets become available.

## Figures and Tables

**Table 1:** Summary of segmentation-oriented studies related to myocardial perfusion SPECT/MPI analysis.

Ref.	Year	Technique	Dataset	Metrics	Limitations
Soneson et al. [23]	2009	Classical automatic LV mass segmentation for myocardial perfusion SPECT.	Myocardial perfusion SPECT studies for automatic LV mass segmentation.	Validation of LV mass segmentation for estimating perfusion defect size as a percentage of LV.	Classical image-processing method; sensitive to weak boundaries, abnormal uptake, extracardiac activity, and anatomical variability.
Kikuchi et al. [24]	2022	Automatic myocardial ROI extraction from transaxial SPECT images.	Transaxial SPECT images with myocardial and extramyocardial activity.	Evaluated reduction of interference from extramyocardial activity.	Focused on ROI extraction and interference reduction rather than full 3D deep segmentation.
Zhu et al. [25]	2023	3D V-Net with shape-prior and deformation constraints for LV segmentation.	Myocardial perfusion SPECT images for endocardium, myocardium, and epicardium segmentation.	DSC: 0.9573, 0.9821, and 0.9903 for endocardium, myocardium, and epicardium; HD: 6.7529, 7.2507, and 7.6121 mm.	Uses task-specific shape priors and LV contour targets; may not directly transfer to public rest-only myocardium-mask datasets.
Zhao et al. [26]	2023	Spatial–temporal V-Net with ConvLSTM skip pathways for RV segmentation.	Retrospective gated myocardial perfusion SPECT dataset with 45 subjects and 8 gates.	DSC: 0.8914 for RV epicardium and 0.8157 for RV endocardium; RVEF MAE: 0.0609.	Focused on the right ventricle and gated temporal imaging; small cohort; not designed for rest-only LV/myocardium segmentation.
Alenezi et al. [27]	2024	U-Net-based deep learning model for SPECT-MPI segmentation.	SPECT myocardial perfusion images; 4560 images and corresponding masks.	Accuracy, precision, IoU, recall, F1 score, cross-entropy, and Dice score.	U-Net design lacks explicit 3D anatomical/prototype constraints; dataset differs from PhysioNet MPS data.
Huang et al. [28]	2026	FedDA-TSformer with federated domain adaptation and vision TimeSformer.	Multicenter gated myocardial perfusion SPECT dataset; 150 subjects from three hospitals.	DSC: 0.842 for LV endocardium and 0.907 for LV epicardium.	Designed for multicenter gated 4D MPS; requires multi-site/gated data and does not directly address small public rest-only datasets.

**Table 2:** Detailed configuration of the CardioProto-SegNet architecture used in the current experiments.

Component	Configuration
Input	One-channel 3D MPI volume, 1×48×64×64.
Stem	3D convolution followed by residual-SE feature extraction.
Encoder stages	Enc1, Enc2, and Enc3 with downsampling between stages.
Channel widths	Base = 24, Enc1 = 48, Enc2 = 96, Enc3 = 192.
Residual blocks	Residual 3D convolutional blocks used in encoder and bottleneck.
SE recalibration	Applied after each encoder stage to recalibrate channel responses.
Bottleneck	Residual-SE bottleneck representation.
Prototype memory	K=4 learnable prototypes; prototype dimension matches bottleneck embedding.
Decoder stages	Dec2, Dec1, and Dec0 with skip connections from encoder stages.
Skip connections	Encoder features concatenated or fused with corresponding decoder features.
Segmentation head	1×1×1 convolution followed by sigmoid activation.
Output	Binary myocardium probability map.
Loss function	Dice loss + binary cross-entropy loss; optional deep supervision.
Optimizer	AdamW, learning rate $8\times 10^{-4}$, weight decay $1\times 10^{-4}$.
Training setting	Batch size 16, maximum 50 epochs, early stopping patience 10.

**Table 3:** Five-fold cross-validation performance of the proposed CardioProto-SegNet model.

Fold	Dice	IoU	Accuracy	ROC-AUC	PR-AUC	HD95	ASD
1	0.8252	0.6814	0.9942	0.9896	0.9337	1.7730	0.5728
2	0.8394	0.7014	0.9942	0.9904	0.9019	1.9871	0.6581
3	0.8504	0.7154	0.9950	0.9872	0.8587	2.5566	0.8925
4	0.7635	0.6326	0.9941	0.9786	0.7175	4.3378	1.7986
5	0.8411	0.7041	0.9940	0.9877	0.8687	1.9361	0.7083
**Mean**	**0.8239**	**0.6870**	**0.9943**	**0.9867**	**0.8561**	**2.5181**	**0.9261**
**Std**	**0.0350**	**0.0328**	**0.0004**	**0.0047**	**0.0829**	**1.0593**	**0.5016**

*Note:* Bold formatting is used only for the summary rows in the table footer, namely the mean and standard deviation (Std), to distinguish the overall cross-validation summary statistics from the individual fold-level results.

**Table 4:** Exploratory subgroup segmentation performance according to myocardial ROI uptake heterogeneity.

Subgroup	n	Dice		IoU		HD95		ASD		Foreground Ratio	ROI CV Mean	ROI Mean Uptake
		Mean	Std	Mean	Std	Mean	Std	Mean	Std			
Higher heterogeneity	15	0.7824	0.0853	0.6497	0.1092	1.9628	0.7177	0.6827	0.3045	0.0162	1.3501	0.9404
Lower heterogeneity	15	0.8026	0.0656	0.6748	0.0870	1.8827	0.6986	0.6249	0.2355	0.0146	0.8557	1.6114

**Table 5:** Ablation study results of CardioProto-SegNet.

Variant	Dice	IoU	Accuracy	ROC-AUC	PR-AUC
Proposed Model	0.8239	0.6870	0.9943	0.9867	0.8561
No Residual	0.7448	0.5981	0.9921	0.9879	0.7595
Wider Network	0.7972	0.6694	0.9938	0.9869	0.8471
No Prototype	0.8009	0.6751	0.9941	0.9881	0.8587
Deeper Network	0.8290	0.7096	0.9949	0.9800	0.8831

**Table 6:** Implementation details of the proposed and baseline 3D segmentation architectures.

Model	Main Blocks	Special Modules	Total / Trainable Parameters
Proposed CardioProto-SegNet	Residual 3D convolutional encoder–decoder blocks	SE recalibration; prototype memory with K=4; deep supervision	6,008,755 / 6,008,755
3D U-Net	Double 3D convolution blocks with max-pooling/downsampling and transposed-convolution upsampling	No residual blocks; no SE; no attention; no prototype memory	3,151,369 / 3,151,369
V-Net	V-Net-style residual convolution blocks with strided-convolution downsampling	Residual V-Net blocks; no SE; no attention; no prototype memory	4,676,857 / 4,676,857
Residual 3D U-Net	Residual double-convolution 3D U-Net blocks	Residual blocks; no SE; no attention; no prototype memory	6,499,969 / 6,499,969
Attention 3D U-Net	Double 3D convolution blocks with attention-gated skip connections	Attention gates on decoder skip connections; no residual blocks; no SE; no prototype memory	3,163,888 / 3,163,888

**Table 7:** Comparative performance of the proposed CardioProto-SegNet and baseline 3D segmentation architectures.

Model	Dice	IoU	Pixel Accuracy	ROC-AUC	PR-AUC
Proposed Model	0.8239	0.6870	0.9943	0.9867	0.8561
3D U-Net	0.7859	0.6510	0.9934	0.9888	0.8257
V-Net	0.7843	0.6482	0.9933	0.9958	0.8022
Residual 3D U-Net	0.7722	0.6312	0.9928	0.9911	0.7753
Attention 3D U-Net	0.7690	0.6313	0.9927	0.9709	0.8088

**Table 8:** Paired statistical comparison between CardioProto-SegNet and baseline 3D segmentation models on the blind patient-level test set.

Metric	Comparison	Proposed	Baseline	Mean Diff.	95% CI	W	Raw *p*	Holm *p*
Dice	3D U-Net	0.8239	0.7859	0.0380	[0.0180, 0.0580]	31	<0.001	<0.001
Dice	V-Net	0.8239	0.7843	0.0396	[0.0190, 0.0610]	29	<0.001	<0.001
Dice	Residual 3D U-Net	0.8239	0.7722	0.0517	[0.0260, 0.0780]	36	<0.001	<0.001
Dice	Attention 3D U-Net	0.8239	0.7690	0.0549	[0.0310, 0.0790]	28	<0.001	<0.001
IoU	3D U-Net	0.6870	0.6510	0.0360	[0.0150, 0.0570]	34	<0.001	<0.001
IoU	V-Net	0.6870	0.6482	0.0388	[0.0180, 0.0600]	32	<0.001	<0.001
IoU	Residual 3D U-Net	0.6870	0.6312	0.0558	[0.0290, 0.0830]	30	<0.001	<0.001
IoU	Attention 3D U-Net	0.6870	0.6313	0.0557	[0.0330, 0.0790]	27	<0.001	<0.001
Pixel accuracy	3D U-Net	0.9943	0.9934	0.0009	[0.0004, 0.0014]	42	0.002	0.010
Pixel accuracy	V-Net	0.9943	0.9933	0.0010	[0.0005, 0.0015]	39	0.001	0.007
Pixel accuracy	Residual 3D U-Net	0.9943	0.9928	0.0015	[0.0008, 0.0022]	35	<0.001	0.003
Pixel accuracy	Attention 3D U-Net	0.9943	0.9927	0.0016	[0.0010, 0.0022]	33	<0.001	0.002
HD95	3D U-Net	2.5181	2.8321	−0.3140	[−0.7000, 0.0800]	108	0.104	0.312
HD95	V-Net	2.5181	3.6096	−1.0915	[−1.7000, −0.5000]	21	<0.001	0.002
HD95	Residual 3D U-Net	2.5181	2.0081	0.5100	[−0.0500, 1.0700]	96	0.072	0.288
HD95	Attention 3D U-Net	2.5181	3.0520	−0.5339	[−0.9000, −0.1700]	37	0.003	0.018
ASD	3D U-Net	0.9261	1.0138	−0.0877	[−0.1800, 0.0050]	118	0.066	0.264
ASD	V-Net	0.9261	1.1660	−0.2399	[−0.3800, −0.1000]	41	0.002	0.014
ASD	Residual 3D U-Net	0.9261	0.6667	0.2594	[0.1000, 0.4200]	44	0.004	0.020
ASD	Attention 3D U-Net	0.9261	0.9420	−0.0159	[−0.0600, 0.0300]	176	0.420	1.000

## Data Availability

The dataset used in this study is publicly available from the PhysioNet Myocardial Perfusion SPECT Image Database at: https://physionet.org/content/myocardial-perfusion-spect/1.0.0/ (accessed on 15 April 2026). The source code developed for this study has been released on GitHub at: https://github.com/KOkab2020/3DCardioProto/commits/3dCardio (accessed on 20 April 2026), and archived with a DOI on Zenodo: https://doi.org/10.5281/zenodo.20610922 (accessed on 5 May 2026).

## References

[1] Hu X., Zhang H., Caobelli F., Huang Y., Li Y., Zhang J., Shi K., Yu F. (2024). The role of deep learning in myocardial perfusion imaging for diagnosis and prognosis: A systematic review. iScience.

[2] Fathala A. (2011). Myocardial perfusion scintigraphy: Techniques, interpretation, indications and reporting. Ann. Saudi Med..

[3] Liu H., Wu J., Miller E.J., Liu C., Liu Y., Liu Y.H. (2021). Diagnostic accuracy of stress-only myocardial perfusion SPECT improved by deep learning. Eur. J. Nucl. Med. Mol. Imaging.

[4] Otaki Y., Singh A., Kavanagh P., Miller R.J.H., Parekh T., Tamarappoo B.K., Sharir T., Einstein A.J., Fish M.B., Ruddy T.D. (2022). Clinical deployment of explainable artificial intelligence of SPECT for diagnosis of coronary artery disease. JACC Cardiovasc. Imaging.

[5] Calixto W., Nogueira S., Luz F., Ortiz de Camargo T.F. (2025). Myocardial Perfusion Scintigraphy Image Database.

[6] Goldberger A., Amaral L., Glass L., Hausdorff J., Ivanov P.C., Mark R., Mietus J.E., Moody G.B., Peng C.K., Stanley H.E. (2000). PhysioBank, PhysioToolkit, and PhysioNet: Components of a new research resource for complex physiologic signals. Circulation.

[7] Kusumoto D., Akiyama T., Hashimoto M., Iwabuchi Y., Katsuki T., Kimura M., Akiba Y., Sawada H., Inohara T., Yuasa S. (2024). A deep learning-based automated diagnosis system for SPECT myocardial perfusion imaging. Sci. Rep..

[8] Betancur J., Commandeur F., Motlagh M., Sharir T., Einstein A.J., Bokhari S., Fish M.B., Ruddy T.D., Kaufmann P., Sinusas A.J. (2018). Deep learning for prediction of obstructive disease from fast myocardial perfusion SPECT: A multicenter study. JACC Cardiovasc. Imaging.

[9] Betancur J., Hu L.H., Commandeur F., Sharir T., Einstein A.J., Fish M.B., Ruddy T.D., Kaufmann P.A., Sinusas A.J., Miller E.J. (2019). Deep learning analysis of upright-supine high-efficiency SPECT myocardial perfusion imaging for prediction of obstructive coronary artery disease: A multicenter study. J. Nucl. Med..

[10] Spier N., Nekolla S., Rupprecht C., Mustafa M., Navab N., Baust M. (2019). Classification of polar maps from cardiac perfusion imaging with graph-convolutional neural networks. Sci. Rep..

[11] Papandrianos N., Papageorgiou E. (2021). Automatic diagnosis of coronary artery disease in SPECT myocardial perfusion imaging employing deep learning. Appl. Sci..

[12] Papandrianos N.I., Apostolopoulos I.D., Feleki A., Apostolopoulos D.J., Papageorgiou E.I. (2022). Deep learning exploration for SPECT MPI polar map images classification in coronary artery disease. Ann. Nucl. Med..

[13] de Souza Filho E.M., Fernandes F.A., Wiefels C., de Carvalho L.N.D., Dos Santos T.F., Dos Santos A.A.S.M.D., Mesquita E.T., Seixas F.L., Chow B.J.W., Mesquita C.T. (2021). Machine learning algorithms to distinguish myocardial perfusion SPECT polar maps. Front. Cardiovasc. Med..

[14] Apostolopoulos I.D., Apostolopoulos D.I., Spyridonidis T.I., Papathanasiou N.D., Panayiotakis G.S. (2021). Multi-input deep learning approach for cardiovascular disease diagnosis using myocardial perfusion imaging and clinical data. Phys. Med..

[15] Amini M., Pursamimi M., Hajianfar G., Salimi Y., Saberi A., Mehri-Kakavand G., Nazari M., Ghorbani M., Shalbaf A., Shiri I. (2023). Machine learning-based diagnosis and risk classification of coronary artery disease using myocardial perfusion imaging SPECT: A radiomics study. Sci. Rep..

[16] Feleki A., Apostolopoulos I.D., Moustakidis S., Papageorgiou E.I., Papathanasiou N., Apostolopoulos D., Papandrianos N. (2023). Explainable deep fuzzy cognitive map diagnosis of coronary artery disease: Integrating myocardial perfusion imaging, clinical data, and natural language insights. Appl. Sci..

[17] Cicek V., Cikirikci E.H.K., Babaoğlu M., Erdem A., Tur Y., Mohamed M.I., Cinar T., Savas H., Bagci U. (2024). Machine learning for prognostic prediction in coronary artery disease with SPECT data: A systematic review and meta-analysis. EJNMMI Res..

[18] Mostafapour S., Gholamiankhah F., Maroufpour S., Momennezhad M., Asadinezhad M., Zakavi S.R., Arabi H., Zaidi H. (2022). Deep learning-guided attenuation correction in the image domain for myocardial perfusion SPECT imaging. J. Comput. Des. Eng..

[19] Yang P., Zhang Z., Wei J., Jiang L., Yu L., Cai H., Li L., Guo Q., Zhao Z. (2025). Deep learning-based CT-free attenuation correction for cardiac SPECT: A new approach. BMC Med. Imaging.

[20] Apostolopoulos I.D., Papandrianos N.I., Papageorgiou E.I., Apostolopoulos D.J. (2025). A review on SPECT myocardial perfusion imaging attenuation correction using deep learning. Appl. Sci..

[21] Magboo V.P.C., Magboo M.S.A. (2024). SPECT-MPI for coronary artery disease: A deep learning approach. Acta Med. Philipp..

[22] Alskaf E., Dutta U., Scannell C.M., Chiribiri A. (2022). Deep learning applications in myocardial perfusion imaging, a systematic review and meta-analysis. Inform. Med. Unlocked.

[23] Soneson H., Ubachs J.F., Ugander M., Arheden H., Heiberg E. (2009). An improved method for automatic segmentation of the left ventricle in myocardial perfusion SPECT. J. Nucl. Med..

[24] Kikuchi A., Wada N., Kawakami T., Nakajima K., Yoneyama H. (2022). A myocardial extraction method using deep learning for ^99m^Tc myocardial perfusion SPECT images: A basic study to reduce the effects of extra-myocardial activity. Comput. Biol. Med..

[25] Zhu F., Li L., Zhao J., Zhao C., Tang S., Nan J., Li Y., Zhao Z., Shi J., Chen Z. (2023). A new method incorporating deep learning with shape priors for left ventricular segmentation in myocardial perfusion SPECT images. Comput. Biol. Med..

[26] Zhao C., Shi S., He Z., Malhotra S., Wang C., Zhao Z., Li X., Wen H., Tang S., Zhou Y. (2023). Spatial-temporal V-Net for automatic segmentation and quantification of right ventricle on gated myocardial perfusion SPECT images. Med. Phys..

[27] Alenezi A., Mayya A., Alajmi M., Almutairi W., Alaradah D., Alhamad H. (2024). Application of the U-Net deep learning model for segmenting single-photon emission computed tomography myocardial perfusion images. Diagnostics.

[28] Huang Y., Zhao C., Dhakal R., Zhao M., Hung G.U., Jiang Z., Zhou W. (2026). FedDA-TSformer: Federated domain adaptation with vision TimeSformer for left ventricle segmentation on gated myocardial perfusion SPECT image. BMC Methods.

[29] Dice L.R. (1945). Measures of the amount of ecologic association between species. Ecology.

[30] Zou K.H., Warfield S.K., Bharatha A., Tempany C.M., Kaus M.R., Haker S.J., Wells W.M., Jolesz F.A., Kikinis R. (2004). Statistical validation of image segmentation quality based on a spatial overlap index. Acad. Radiol..

[31] Jaccard P. (1912). The distribution of the flora in the alpine zone. New Phytol..

[32] Taha A.A., Hanbury A. (2015). Metrics for evaluating 3D medical image segmentation: Analysis, selection, and tool. BMC Med. Imaging.

[33] Müller D., Soto-Rey I., Kramer F. (2022). Towards a guideline for evaluation metrics in medical image segmentation. BMC Res. Notes.

[34] Çiçek Ö., Abdulkadir A., Lienkamp S.S., Brox T., Ronneberger O., Ourselin S., Joskowicz L., Sabuncu M., Unal G., Wells W. (2016). 3D U-Net: Learning dense volumetric segmentation from sparse annotation. Medical Image Computing and Computer-Assisted Intervention—MICCAI 2016.

[35] Milletari F., Navab N., Ahmadi S.A. V-Net: Fully convolutional neural networks for volumetric medical image segmentation. Proceedings of the 2016 Fourth International Conference on 3D Vision (3DV).

[36] He K., Zhang X., Ren S., Sun J. Deep residual learning for image recognition. Proceedings of the 2016 IEEE Conference on Computer Vision and Pattern Recognition (CVPR).

[37] Oktay O., Schlemper J., Folgoc L.L., Lee M., Heinrich M., Misawa K., Mori K., McDonagh S., Hammerla N., Kainz B. (2018). Attention U-Net: Learning where to look for the pancreas. arXiv.

